# Assessment of Multiple Ecosystem Services and Ecological Security Pattern in Shanxi Province, China

**DOI:** 10.3390/ijerph20064819

**Published:** 2023-03-09

**Authors:** Jinfeng Wang, Ya Li, Sheng Wang, Qing Li, Lingfeng Li, Xiaoling Liu

**Affiliations:** 1 School of Geographical Science, Shanxi Normal University, Taiyuan 030031, China; 2 Institute of Geographical Sciences, Hebei Academy of Sciences, Hebei Engineering Research Center for Geographic Information Application, Shijiazhuang 050011, China

**Keywords:** ecosystem services, ecological security pattern, hotspot analysis, minimum cumulative resistance

## Abstract

The ecological security pattern construction could effectively regulate ecological processes and ensure ecological functions, then rationally allocate natural resources and green infrastructure, and, finally, realize ecological security. In view of serious soil erosion, accelerated land desertification, soil pollution and habitat degradation in Shanxi Province, the spatial distribution of six key ecosystem services, including water conservation (WC), soil conservation (SC), sand fixation (SF), carbon storage (CS), net primary productivity (NPP) and habitat quality (HQ), was analyzed by using multiple models. The comprehensive ability of multiple ecosystem services in different regions was quantified by calculating multiple ecosystem services landscape index (MESLI). Combined with ecosystem services hotspots, the ecological security pattern of Shanxi Province was constructed by using the minimum cumulative resistance model. The results showed that the spatial differences in ecosystem services in Shanxi Province were obvious, which was low in the seven major basins and Fen River valley, and high in the mountains (especially Taihang and Lvliang Mountains) for WC, SC, CS, NPP and HQ, while high SF was only distributed in the northern Shanxi. The MESLI showed that the ability to provide multiple ecosystem services simultaneously was low in Shanxi Province, with the medium and low grade MESLI regions accounting for 58.61%, and only 18.07% for the high grade MESLI regions. The important protected areas and ecological sources of the ecological security pattern were concentrated in the Lvliang and Taihang Mountains, which were consistent with the key areas of ecosystem services. The ecological corridors illustrated network distribution with ecological sources as the center, the low-, medium- and high-level buffers accounted for 26.34%, 17.03% and 16.35%, respectively. The results will provide important implications for economic transformation, high-quality development and ecological sustainable development in resource-based regions worldwide.

## 1. Introduction

In the complex social-ecological system, both human well-being and social sustainability depend on ecosystem services (ESs, all terms and abbreviations in File S1) provided by nature [1,2]. The combination of ecosystem regulation, supply, support and culture services will contribute to achieve the synergistic goals of sustainable development [3,4]. In the context of population growth, social and economic development, and intensified contradiction between human and land, how to enhance ecosystem services to maximize the human welfare has become the current focus of social-ecological sustainability [5,6]. Recent studies are no longer limited to conceptual analysis [1,7] or single indicators [8,9]; comprehensive research on multiple ecosystem services and their relationships provide important guidelines for global and regional ecological civilization construction [10,11,12].

At present, unreasonable planning leads to frequent regional ecological security issues, such as the health and sustainability of ecosystem services, resources and environment, have become the important part of ecological protection [13,14]. The assessment of ecological security pattern could identify the ecological network composed of different landscape elements, strengthen the connectivity among landscape elements, better protect natural resources and ecological well-being, and thus provide scientific basis for maintaining regional ecological security and optimizing ecological environment planning [15,16]. The Minimum Cumulative Resistance (MCR) model is an effective method for building ecological security pattern. Based on the MCR model, a typical research paradigm of “ecological source—resistance surface—corridor extraction—ecological security pattern” was established [17,18]. By combining the MCR model with Duranton–Overman Index (DOI), Dai et al. [19] assessed the ecological security network for the urban agglomeration around Poyang Lake in China, solved the spatial conflict between ecological protection and economic development of the urban agglomeration and effectively avoided the threat of industrial layout to the ecological landscape. Wang et al. [20] analyzed the spatial and temporal variation of four key ecosystem services (WC, SC, NPP and HQ), quantified the impact of different climate factors and land use change on ecosystem services and constructed ecological security pattern by using the MCR model in the Beijing-Tianjin-Hebei region. At present, scholars pay more attention to biodiversity conservation, ecosystem maintenance and landscape integrity, and are less concerned with the relationship between ecosystem service and ecological space, and their impacts on regional ecological security [21]. The identification of ecological sources is the key to ecological security patterns construction [22]. In previous studies, ecological sources were usually determined by forest land, nature reserves, ecological red lines and ecosystem service values [23,24], and few were based on multiple ecosystem services hotspots and connectivity analysis. Due to the vast territory and diverse ecosystem types, the response of ecosystem functions and processes to the natural and socio-economic changes are much different. Thus, a comprehensive quantitative indicator, combined with the indexes of ecosystem services and regional ecological background, could effectively identify the ecological sources and better build the local ecological security pattern.

In recent decades, the negative impact on environment caused by urban expansion in the Yellow River Basin has become increasingly prominent, and the regional ecology is more fragile due to the contradiction between natural environment conservation and urban development [25,26]. Accompanied by the concept of ecological protection and high-quality development in the Yellow River Basin, how to realize the coordinated development of ecological security and social economy has become a hot issue [27]. Shanxi Province is located in the Loess Plateau in the middle reaches of the Yellow River Basin, with soil erosion area as high as 10.8 × 10^4^ km^2^, accounting for 69% of the total area [28]. The northern Shanxi is located in the agro-pastoral ecotone, which is one of the strongest soil wind erosion regions in China. Further, Shanxi Province is an important coal energy-steel industry base in China. In recent years, with the rapid development of long-term coal mining, urbanization and industrialization, multiple environmental problems, such as the land use change, the serious destruction of vegetation and soil internal structure and the reduction in biomass and productivity, have accelerated the habitat degradation in Shanxi Province [29]. Grassland degradation, farmland reclamation and urban expansion greatly reduced sand fixing service of ecosystem [30]. Land desertification has expanded southward at a speed of 10 m/a, sandstorms and dust-floating weather occurred frequently [31]. In addition, the carbon sink function of soil carbon pools (carbon storage accounts for more than 90% in terrestrial ecosystems) strongly declined due to soil pollution, erosion and other problems [32,33]. At the same time, the coal-steel industrial system depends on the high coal consumption, and the pressure of energy conservation and emission reduction is increasing [34]. Therefore, the deep-seated contradictions in the development of resource-based economy are becoming increasingly prominent, which seriously restrict the high-quality and sustainable development of social economy in Shanxi Province.

Under the influence of global ecosystem changes, the coordinated development of natural elements has become the primary task for maintaining ecological security. The construction and management of ecological security pattern can provide scientific guidance for large-scale land spatial planning and, thus, reduce the local ecologically land and habitat fragmentation caused by urbanization disorder expansion [35]. Shanxi Province, which is the first provincial comprehensive reform demonstration zone in China, is undergoing the transition from traditional to green economy [36]. Both environmental protection and ecological restoration are important tasks in economic transformation. It is urgent to comprehensively assess the integrity and health of ecosystems by ascertaining the status of regional ecosystem services and building ecological security patterns. However, few studies on ecosystem services were carried out in Shanxi Province; only a few case studies assessed the ecosystem services [37], or preliminarily built the ecological security pattern in the catchment scales [38]. In addition, scholars also paid attention to ecological restoration, assessed the importance of ecosystem services and constructed the ecological compensation systems in coal mining areas. In general, some deficiencies are as follows. (1) Due to the lack of comprehensive research on Shanxi Province, it is difficult to evaluate the applicability of ecological protection policies and the effectiveness of regional ecological restoration projects. (2) Only a few indicators (such as soil and water conservation) were selected to evaluate the regional ecosystem services, lacking a suitable indicator and model system for ecosystem service assessment in Shanxi Province. Therefore, the specific objectives of this study were to: (1) analyze the spatial distribution of six key ecosystem services (water conservation, soil conservation, sand fixation, carbon storage, net primary productivity and habitat quality) in different regions; (2) quantify the comprehensive ability of multiple ecosystem services in Shanxi Province by calculating MESLI; and (3) construct the ecological security pattern applicable to Shanxi Province by combining ecosystem services hotspots. This study will not only provide scientific guidance for ecological security protection, comprehensive restoration and management of regional ecosystem in Shanxi Province, but also have reference value for the construction of ecological security pattern and sustainable development of ecosystem in the same resource-based regions and ecologically fragile regions in the world.

## 2. Materials and Methods

### 2.1. Study Area

**Figure 1:**
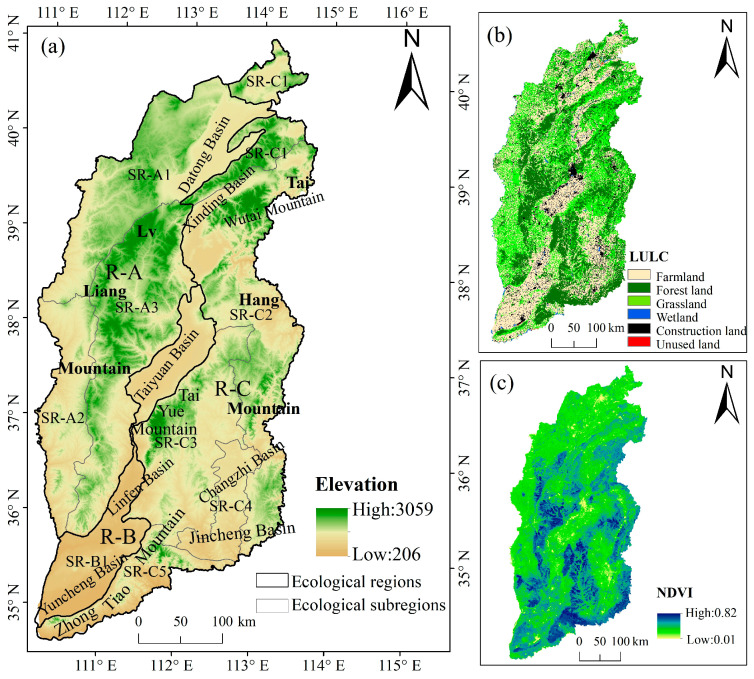
(**a**) Location of the study area; (**b**) spatial distribution of land use/land cover; (**c**) spatial distribution of NDVI.

Shanxi Province is located in the Loess Plateau (34.34° N–40.44° N, 110.14° E–114.33° E, Figure 1), with a total area of 15.67 × 10^4^ km^2^ [39]. More than 80% are mountains and hills, and the plains are in the intermountain valley areas. Shanxi Province is the temperate continental monsoon climate, with annual average temperature of 3.4–14.7 °C, annual average precipitation of 554 mm. Under the influence of climate, the vegetation type in the south is mainly deciduous broad-leaved forest and coniferous broad-leaved mixed forest; the north is covered by temperate scrub and semi-arid grassland, which is an important part of the sandstorm source control project. The study area is divided into three ecological regions and nine ecological subregions (Table 1) according to the ecological zoning from the Research Center for Eco-Environmental Sciences, Chinese Academy of Sciences (http://www.ecosystem.csdb.cn, accessed on 2 December 2021), and natural conditions, such as topography, climate, vegetation and ecosystem structure of Shanxi Province.

### 2.2. Data Sources

The materials required in this study include meteorological, remote sensing, water resources and sediment volume data. The meteorological data were obtained from the China Meteorological Data Service Centre (http://data.cma.cn, accessed on 3 February 2022), including the daily average temperature, precipitation, solar radiation and potential evapotranspiration of 76 meteorological stations in and around Shanxi Province in 2020, and were interpolated into month-by-month raster data with spatial resolution of 1 km × 1 km by AUSPLINE software. The hourly wind speed data of 56 stations around Shanxi Province in 2020, and the cumulative time of each grade wind speed, which is greater than or equal to the critical erosion wind speed (5 m s^−1^) during the wind erosion activities, were obtained from the National Climatic Data Center (NCDC, ftp://ftp.ncdc.noaa.gov/pub/data/noaa/isd-lite/, accessed on 20 February 2022). The remote sensing data included land use, normalized difference vegetation index (NDVI), soil, digital elevation model (DEM) and Chinese road and river network vector data. The land use/land cover (LULC) data of Shanxi Province in 2020 were downloaded from the Resource and Environment Science Data Center, Chinese Academy of Sciences (http://www.resdc.cn/, accessed on 15 March 2022), with the spatial resolution of 100 m × 100 m. The NDVI data was downloaded from National Aeronautics and Space Administration (NASA, https://search.earthdata.nasa.gov/search, accessed on 7 May 2022), with a resolution of 250 m × 250 m. The soil data (1: 1,000,000) were from the Institute of Soil Science, Chinese Academy of Sciences, including soil type maps and soil attribute data. The DEM was ASTER GDEM data, with the resolution of 30 m × 30 m, and was obtained from the NASA Data Distribution Center (http://gdem.ersdac.jspacesystems.or.jp/, accessed on 15 March 2022). The Chinese road and river network vector data were obtained from the Resource and Environment Science Data Center, Chinese Academy of Sciences (https://www.resdc.cn/, accessed on 9 December 2021). The water resources and sediment volume data were obtained from the Shanxi Water Resources Bulletin in Shanxi Provincial Department of Water Resources (http://slt.shanxi.gov.cn/, accessed on 28 December 2021).

### 2.3. Methods

#### 2.3.1. Ecosystem Service Estimation

Based on the ecological problems faced by Shanxi Province, such as soil erosion, accelerated land desertification, soil pollution, habitat degradation, and destruction of forest and grass vegetation, six important ecosystem services, including water conservation (WC), soil conservation (SC), sand fixation (SF), carbon storage (CS), net primary productivity (NPP) and habitat quality (HQ), were selected to build an indicator system by using Integrated Valuation of Ecosystem Services and Tradeoffs (InVEST), Carnegie Ames Stanford Approach (CASA) and National Wind Erosion Survey Model of China (NWESMC). The model descriptions are shown in Table 2 and File S2.

#### 2.3.2. Multiple Ecosystem Services Landscape Index (MESLI)

The Multiple Ecosystem Services Landscape Index (MESLI) could identify the comprehensive ability of multiple ecosystem services in different regions [49]. MESLI is defined as the sum of standardized ecosystem services indicators, which is derived from the weighted average of six standardized ecosystem services, including WC, SC, SF, CS, NPP and HQ [50]. The weight of each ecosystem service is determined by principal component analysis. The effectiveness and applicability of principal component analysis is tested through the Kaiser–Meyer–Olkin (KMO) method [51]. The KMO index was 0.477 (KMO < 0.5), which could not pass the principal component analysis test, indicating that there was no statistical primary and secondary difference among the above six indicators; thus, the weights were all assigned as 1/6.

#### 2.3.3. Hotspot Analysis

For the spatial distribution of six ecosystem services in Shanxi Province, the grids, whose ecosystem services exceeded the respective average values of all grids, were defined as hotspots of this kind of ecosystem services [20]. Hotspots were divided into seven classes from 0 to 6 by overlaying analysis of the six ecosystem services hotspots. If only one ecosystem service of a certain grid was above the average, the grid was defined as class 1 hotspot. Similarly, class 2, 3, 4, 5 and 6 hotspots were defined. If none of the ecosystem services of the grids exceeded the corresponding average value, the grids were defined as non-hotspot area [52].

#### 2.3.4. Ecological Security Pattern Construction

Ecological sources, corridors and buffers are the main components of ecological security patterns [17]. Ecological sources, which are usually located in areas with well-connected landscape patterns and high ecosystem service values, are of great significance for the maintenance and dispersal of species [53]. In this study, the ecological sources were comprehensively delineated through hotspots and landscape connectivity of six ecosystem services.

**Figure 2:**
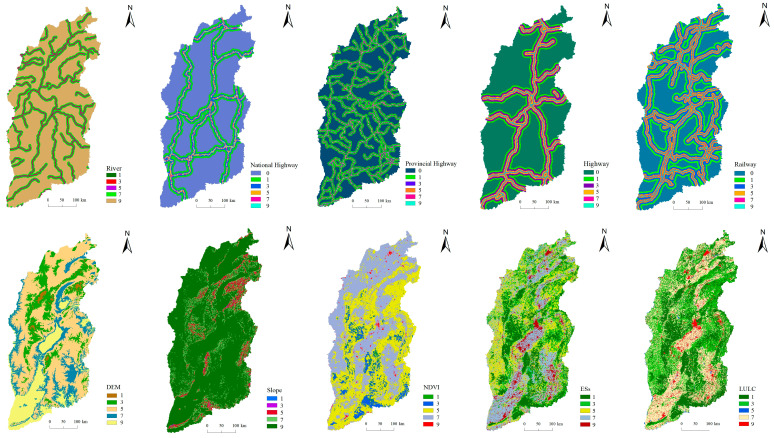
Construction of single ecological resistance surface in Shanxi Province.

The resistance surface reflected the potential possibility and trend of species movement. According to the current ecological environment in Shanxi Province, 10 resistance factors of elevation, slope, ecosystem services, land use, vegetation cover, distance from river, distance from national highway, distance from provincial highway, distance from highway and distance from railway were selected for resistance surface construction from the perspective of nature, economy and society (Figure 2) [54,55]. The analytic hierarchy process (AHP) was used to determine the weight of each resistance factor for ecological source (Table S7) and urban land (Table S8). The spatial weighting was calculated in GIS platform to obtain the resistance surfaces in the expansion process of ecological sources and urban in Shanxi Province.

The ecological corridor was defined as the minimum cost distance from the resistance surface to the two adjacent ecological sources [56,57], which was identified by the minimum cumulative resistance (*MCR*) model. The formula was as follows:

(1) $$MCR=f\min\sum_{j=n}^{i=m}D_{ij}R_{i}$$

where *MCR* is the minimum cumulative resistance, *f* is a positive correlation function between *MCR* and ecological process, *D_ij_* represents the spatial distance from source *j* to landscape unit *i*, and *R_i_* represents the resistance coefficient of landscape unit *i* to species movement.

The final *MCR* (*MCR_diff_*) was controlled by both ecological sources expansion and urban expansion, which was defined as the difference between the minimum cumulative resistance of ecological source expansion and urban land expansion [58,59]. The *MCR_diff_* was calculated as follows:

(2) $$MCR_{diff}=MCR_{ES}-MCR_{UL}$$

where *MCR_ES_* and *MCR_UL_* are the minimum cumulative resistance of ecological source expansion and urban land expansion, respectively. The *MCR_diff_* < 0 represents the area suitable for ecological sources expansion, *MCR_diff_* > 0 represents the area suitable for urban land expansion and *MCR_diff_* = 0 represents the dividing line between the ecological source and urban land expansion area.

## 3. Results

### 3.1. Spatial Pattern of Ecosystem Services

**Figure 3:**
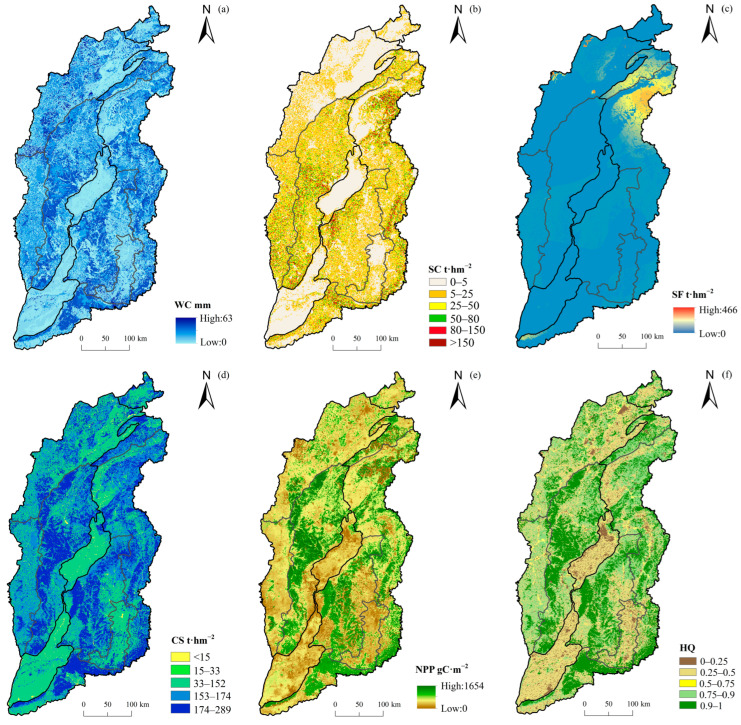
Spatial pattern of six ecosystem services ((**a**) WC, (**b**) CS, (**c**) SF, (**d**) SC, (**e**) NPP and (**f**) HQ) in Shanxi Province.

The spatial patterns of WC, SC, SF, CS, NPP and HQ in Shanxi Province were shown in Figure 3. For WC, SC, CS, NPP and HQ (Figure 3a–b,d–f), the spatial distributions were high in the western Lvliang Mountains and the eastern Taihang Mountains extending from northeast to south. The ESs were low in the central Datong basin, Taiyuan basin, Fen River valley, Changzhi basin and Jincheng basin from north to south, with the annual average of 13.8 mm, 21.7 t·hm^−2^, 180.9 t·hm^−2^, 629.7 gC·m^−2^, and 0.64, respectively. However, SF (Figure 3c) was distributed in the northeast and northwest of Shanxi Province, with an average of 3.7 t·hm^−2^.

For the six ecosystem services under different land use types (Table 3), the average value of six ecosystem services in forest land was 1.29–1.63 times of Shanxi Province, especially NPP, which was 399.4 gC·m^−2^ higher than the average value in the study area. The ecosystem service of grassland was second, and the SF was 1.79 times of the average value of Shanxi Province. However, the ecosystem service in construction land was poor; except for WC and NPP, the ecosystem services were all less than 50% of the average value in the study area. On the whole, the comprehensive capacity of six ecosystem services under different land use types was forest land > grassland > farmland > wetland > unused land > construction land.

There were significant differences in six ecosystem services of different ecological regions and subregions (Table 4). The ecosystem service of R-C was the highest, which was 1.05–1.87 times of the average value in Shanxi Province. R-A came second, and R-B was poor. The SF was only 0.1 t·hm^−2^ in R-B, which was 3% of the average value in the study area. The dominant ecosystem services in each ecological subregion were different. The most important area of SC was mainly distributed in SR-A2 and SR-C5, with the area of 2.07×10^4^ km^2^, and the dominant land use types were forest land and grassland, accounting for 64% of the subregion area. The most important areas for WC, CS, NPP and HQ were located in SR-A3, SR-C5 and SR-C3, and the dominated land use types were farmland and forest land. The significant region of SF was distributed in the SR-C2 and SR-C1, with a total area of 3.87×10^4^ km^2^, was dominated by grassland, accounting for 37% of the total grassland in Shanxi Province. The ecosystem services both in SR-C4 and SR-B1 were poor, which were mainly farmland, accounting for 58% and 71% of the subregion area, respectively.

### 3.2. Spatial Distribution of MESLI

**Figure 4:**
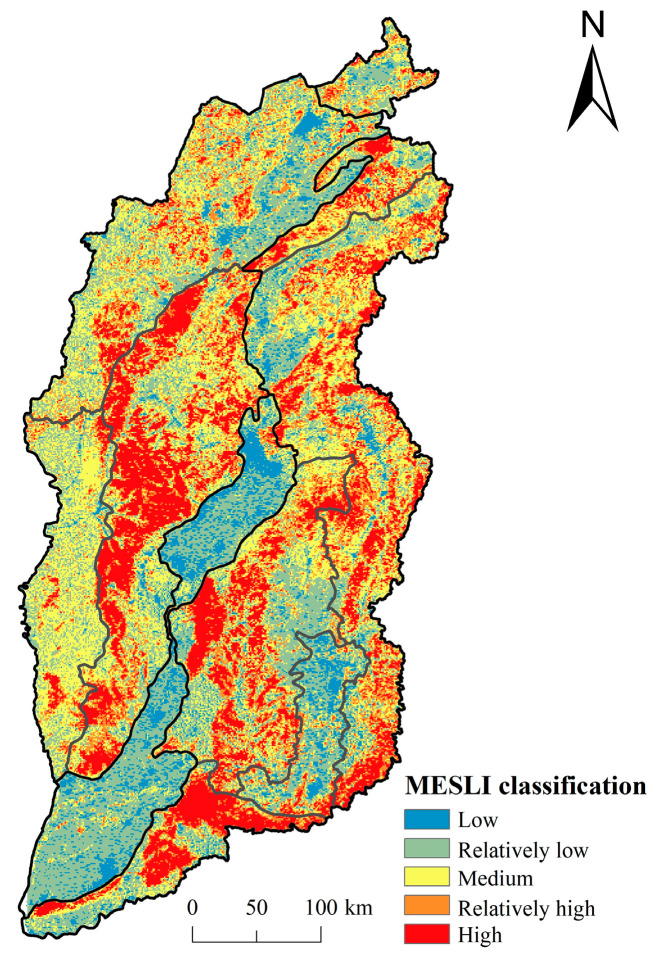
Spatial distribution of MESLI classification in Shanxi Province.

As shown in Figure 4, the MESLI in Shanxi Province was high in the east-west side and low in the middle, presenting obvious spatial heterogeneity. The MESLI was high in mountainous area, while relatively low in the seven basins (Datong basin, Xinzhou basin, Taiyuan basin, Linfen basin, Yuncheng basin, Changzhi basin and Jincheng basin). Most areas in Shanxi Province showed low and medium MESLI, accounting for 58.61%. The low MESLI area (29.48%) was covered by farmland, accounting for more than 70%. The medium MESLI area (29.13%) was dominated by grassland (52.11%), which was mainly distributed in SR-C2 and SR-A1. The high MESLI area accounted for 18%, which concentrated in forest land and grassland of the mountainous areas (SR-A3, C2, C3 and C5), with more than 97% forest land in Shanxi Province. In addition, the low MESLI area was only 7.8%, and was concentrated in urban and rural areas with intensive human activities, which was most closely related to human well-being and urgently needed to upgrade ecosystem services.

### 3.3. Hotspot Analysis of Ecosystem Services and Spatial Distribution of Ecological Sources

**Figure 5:**
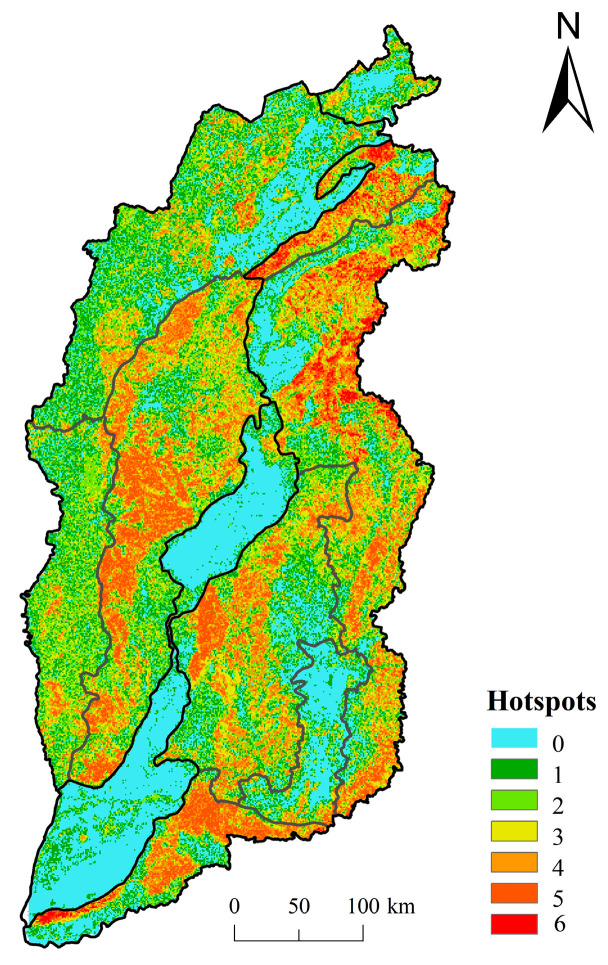
Spatial distribution of key ecosystem service hotspots classification in Shanxi Province.

According to the spatial distribution of key ecosystem services hotspots (Figure 5, Table S9), the class 0~1 ecosystem services hotspots in Shanxi Province accounted for more than 43%, concentrating in the central and eastern basins. The proportion of class 6 ecosystem services hotspots was only 1.28%, mainly dispersed in the northeast of Taihang Mountain. In terms of different land use types, 85% of the construction land and 73% of the farmland were distributed in the class 0 and class 1 ecosystem services hotspots. The ecological land, such as forest land and grassland, provided high quality ecosystem services (at least two types), with 71% of the forest land in the class 4~6 ecosystem services hotspots, and 61% of the grassland in the class 2~4 ecosystem services hotspots.

**Figure 6:**
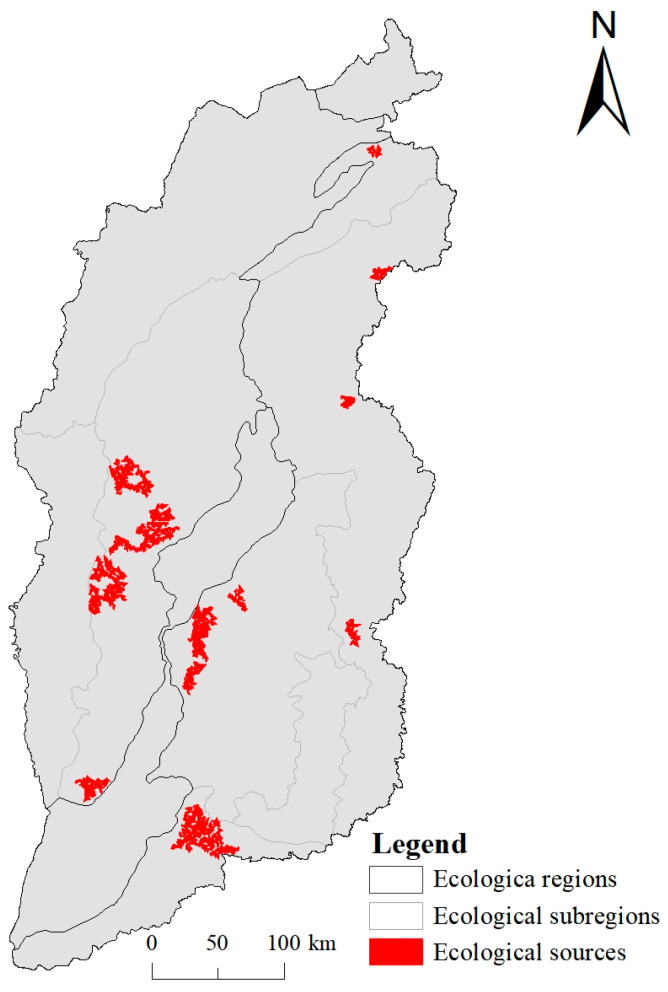
Spatial distribution of ecological sources in Shanxi Province.

Seven classes (0–6) were obtained through the hotspot zoning of ecosystem services, and the ecological sources were identified from the class 5–6 hotspots. In order to reduce the fragmentation of the ecological source areas, the patches with an area of >50 km^2^ were selected to form the final ecological sources. As shown in Figure 6, 16 ecological sources were identified by combining ecosystem services hotspots (class 5–6) with patch connectivity, with the total area of 3094 km^2^. The ecological sources were concentrated in the central and southern Lvliang Mountains, the western Taiyue Mountains and the northern Zhongtiao Mountains, and also scattered in the Sanggan River basin and Taihang Mountains in northern Shanxi. Further, the ecological sources were mainly forest land and grassland (accounting for more than 50% of the total area); in particular, in the central Taihang Mountains, the proportion was up to 71%.

### 3.4. Construction of Ecological Security Pattern

**Figure 7:**
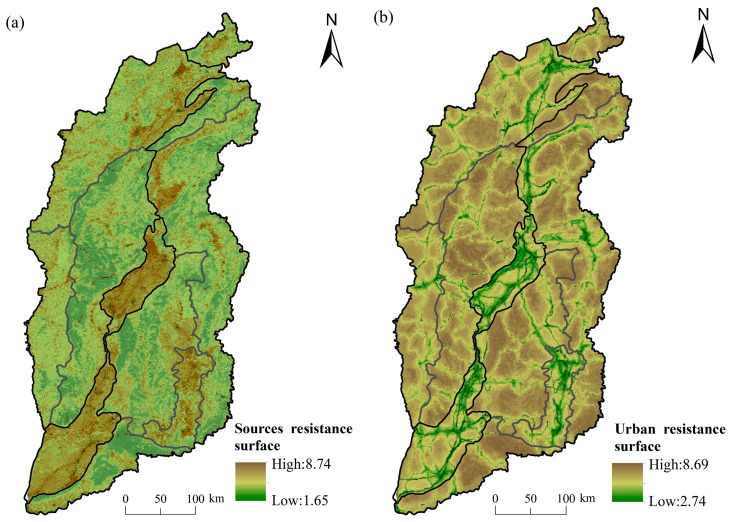
Comprehensive expansion resistance surface of (**a**) ecological sources and (**b**) urban construction in Shanxi Province.

The comprehensive resistance surface was constructed by overlaying single resistance surface. The resistances of ecological sources were low in mountainous areas, including the central Lvliang Mountains, western Taiyue Mountains, northern Zhongtiao Mountains and Taihang Mountains. However, the high resistances were distributed in the central basin and Changzhi basin to the southeast, with rivers, superior topography, convenient transportation and frequent human activities, which have great impact on the ecological environment (Figure 7a). The resistances of urban construction were low in the seven basins and surrounding areas, while high in the mountainous areas (Figure 7b).

**Figure 8:**
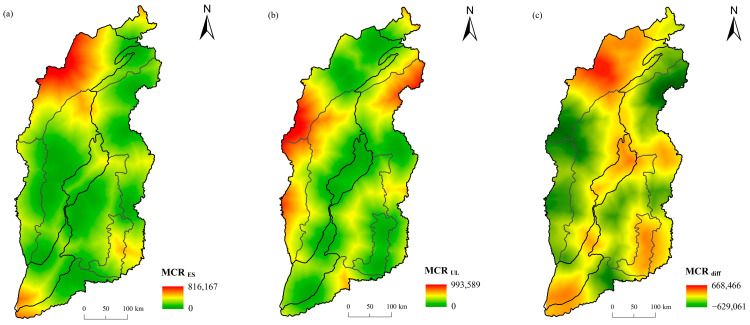
Distribution of minimum cumulative resistance surface for (**a**) ecological sources expansion and (**b**) urban expansion, and (**c**) the MCR difference in Shanxi Province.

Using the ecological sources and the urban land as source data, the comprehensive resistance surfaces of the ecological source expansion and the urban land expansion as consumption distance data, the minimum cumulative resistance value of the ecological source expansion and the urban expansion, and the difference were calculated, respectively (Figure 8). The *MCR_diff_* in Shanxi Province ranged from −629061 to 668446, with high values mainly in the northwest, the central Fenwei River Valley and the southeast basin, with low values mainly in the Lvliang Mountains, Taihang Mountains, Taiyue Mountains and Zhongtiao Mountains. In fact, the minimum accumulation value is not only the spatial resistance value, but also reflects the selectivity of the sources for habitat and the disturbance degree of landscape to species.

**Figure 9:**
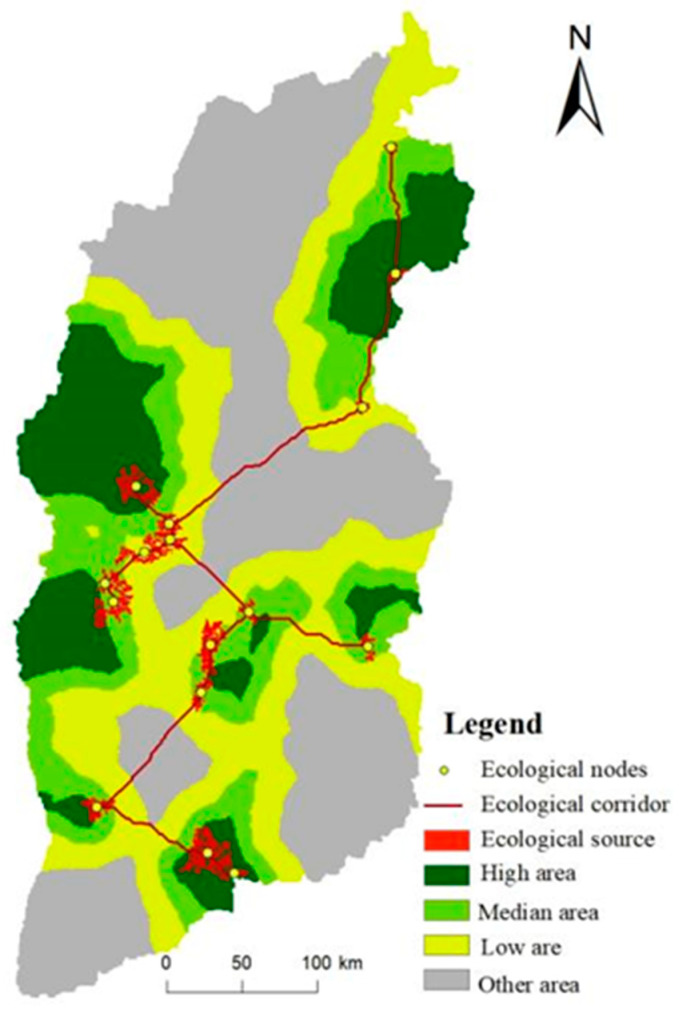
Ecological security pattern of Shanxi Province.

Fifteen major ecological corridors identified by the MCR model, with the total length of 1000 km, formed the network layout and increased the spatial connectivity among ecological sources in Shanxi Province (Figure 9). The ecological corridors were in the shape of “X” and attached the key ecological sources, forming the “three horizontal and two vertical” corridor axes. The “three horizontal” axes connected Taihang Mountain–Xinzhou basin–Lvliang Mountain, Lvliang Mountain–Fen River valley–Taiyue Mountain–Taihang Mountain, Huoyan Mountain–Yuncheng basin–Zhongtiao Mountain. The “two vertical” axes attached the northeast Shanxi–Lvliang mountain, Taiyue mountain–southern Shanxi. The corridors in the central and southern Shanxi Province were highly networked, which were conducive to the mutual diffusion of species, energy and ecology among ecological sources. However, the energy flow, material exchange and species migration among ecological sources were limited in the northwest region without ecological corridors.

Three ecological security pattern buffers (low, medium and high) were obtained by dividing the boundary for the expansion cost of ecological sources (Figure 9). Low-level buffer was the key ecological protection area, with an area of 4.13 × 10^4^ km^2^ (26% of the total area), showing zonal distribution in Lvliang Mountain, Taiyue Mountain, Taihang Mountain and Zhongtiao Mountain. High-level buffer was 2.56 × 10^4^ km^2^ (16%), which was the ideal area for proper development and construction. Medium-level buffer was 2.67 × 10^4^ km^2^ (17%), which was the transition area between low- and high-level buffers.

## 4. Discussion

### 4.1. Ecological Corridors Optimization

The ecological corridor network needs to be optimized to improve the landscape pattern in northwest Shanxi. The northwest Shanxi is the necessary route for northern sandstorm to invade Beijing and Tianjin, so the ecological corridor of Pianguan River–Guancen Mountain–Hutuo River–Luya Mountain–Xizhou Mountain should be added to connect the north–south ecological resources, and the shelterbelts along Lanyi River–Zhujiachuan River–Pianguan River should be established to protect the internal ecological connectivity in the northwest. Considering the destruction on ecological corridors by surface and underground coal mines (Datong Coalmine, Xishan Coalmine, Huoxi Coalmine, Qinshui Coalmine), the phased mining plan for each mining industry is needed to coordinate mining intensity and ecological restoration. The bridges and underground passages for biological migration can be built, and the three-dimensional greenway space can also be constructed by combining the capital flow and natural environment conditions. In addition, the green belt for ecological restoration should be planned around various coal mines to improve the surrounding environment for biological passage, and there should be ongoing development and expansion of the shelterbelts in the northern sandstorm-stricken areas.

Restoring ecological corridors and assessing ecosystem services are efficient measures to protect global biodiversity [60,61,62]. As global population increases, natural land cover is decreasing and most forms of ecological degradation have an overwhelmingly negative effect on biodiversity [63]. The rapid conversion of forests (such as Amazon Rain Forest [64,65] and Caribbean Forest [66]) for agriculture, timber production and other uses has generated vast landscapes dominated by humans, with potentially dire consequences for local biodiversity. Tallgrass prairie once covered large areas of the continental United States, while the expanded agriculture since the 20th century has led to serious degradation of grassland ecosystem [67,68]. As a classic case, the optimization scheme of ecological corridors based on ecosystem service assessments in Shanxi has global applicability to the regions with abundant biological resources and fragile environment, especially the areas with intense human activities. It not only provides scientific support for local ecological restoration, but also presents important reference for global construction of ecological corridors and biodiversity conservation.

### 4.2. Planning Strategies under the Ecological Security Pattern

The ecological security pattern is constructed to protect the ecological barrier and maintain the natural ecosystem structure integrity. To clarify the spatial distribution of forest (shelterbelts in northern Shanxi, forests in Lvliang and Taihang Mountains), farmland (Xinding, Linfen and Yuncheng basin), grass (Lvliang and Taihang Mountains) and water (Fen River, Yellow River), the spatial developments of Shanxi Province should be carried out in “five key zones” based on the ecological security pattern. The Hengshan Mountain in northern Shanxi is sand fixation service area. The NPP, CS and HQ services are concentrated in the Lvliang Mountain (western Shanxi) and Taihang Mountain (eastern Shanxi). The ecosystem in the Taiyue Mountain (central Shanxi) and Zhongtiao Mountain (southern Shanxi) provided the water and soil conservation services.

The ecological sources and low-level buffers within the “five key zones” are given priority protection, and the interference of economic development and human activities are prohibited. Ecological projects, such as nature reserves and national parks, can be appropriately developed in the medium-level buffers. The ecological corridors that were coordinated with ecological sources should be added in the northwest Shanxi to improve the regional ecological security. In the high-level buffers, it should actively play the role of economic development in promoting ecological civilization. Through coordinating the relationship among beautiful environment, touristic ecology and high-quality economy, the transformation and upgrade of resource-based cities and sustainable green development will eventually be realized.

At present, more and more attention is being paid to the impact of environmental change on ecosystem services worldwide. For example, Osland et al. [69] reported that mangrove range expansion was expected to increasingly affect wetland stability and multiple key ecosystem services in the southeastern USA, with ecological trade-offs among different ESs. Mengist et al. [70] found that the Kaffa biosphere reserve in southwest Ethiopia has the potential to release significant amount of emitted carbon. Previous studies lacked the understanding of ecological security pattern for key regions, and there was difficultly in putting forward targeted measures for local ecological protection and restoration. In this study, “five key regions” were divided based on the dominant ecosystem services, and the MCR model was applied to construct the local ecological security pattern. The ecosystem “function-structure” conceptual framework and ecological security classification have global applicability, which provided a classic paradigm for the studies on ecological security and ecological sustainability.

### 4.3. Limitations and Prospects

The global food security is under severe pressure due to extreme weather, disasters and wars, etc. [71,72]. The Russian–Ukrainian war triggered a tsunami that dramatically impacted the world economy, geopolitics, and food security [73]. Food supply is an important component of ecosystem service system [1,7]. In Shanxi Province, farmland in Yuncheng Basin and Changzhi Basin accounts for more than 50%, and the regional food supply is significantly more than other regions. Recently, tourism and culture are increasingly attractive with the improvement of material living standard and spiritual pursuit [74]. Many post-industrial regions (e.g., the Ruhr region of Germany) reinterpret their industrial past as a heritage resource [75]. Industrial heritage is utilized for both immediate tourism marketing purposes and as a tool for memory and identity politics. As a region with an energy-steel economy, Shanxi has similar abundant industrial heritage. Meanwhile, Shanxi has famous natural tourist attractions, such as Wutai Mountain, Taihang Mountain Grand Canyon, Yunqiu Mountain and Hukou Waterfall, as well as national nature reserves, such as Luya Mountain and Lingkong Mountain. In order to guide local ecological protection and restoration, this study pays more attention to regulation and support services of ecosystems. However, on the premise of not damaging the environment, the reasonable utilization of natural resources can realize the coordinated development between regional economy and nature, which should consider supply and culture services. Therefore, our future work is to improve the index system of ecosystem services by integrating support, regulation, supply and culture services. In addition, this study has analyzed the spatial distributions of ecosystem services; a long-term quantitative assessment is needed to clarify the dynamic process of ecosystem.

## 5. Conclusions

As a region with intense human activities and rich resources, Shanxi Province is faced with serious ecological problems, such as soil erosion, desertification, vegetation destruction and habitat degradation. In this study, six key ecosystem services were analyzed and used to construct a regional ecological security pattern in Shanxi Province. The spatial pattern of ecological security was divided into “five key zones”. The NPP, CS and HQ services were low in the central basin (SR-B) and high in the Taihang Mountain (SR-C2) and Lvliang Mountain (SR-A3); the area with high SF was only distributed in the Hengshan Mountain (SR-C1); and the high WC and SC were located in the Taiyue (SR-C3) and Zhongtiao Mountain (SR-A5), which were the ecological sources and critical areas of ecosystem service and ecological security. The regions with high MESLI accounted for 18%, indicating poor ability to provide multiple ecosystem services simultaneously. The ecological corridors were in the shape of “X” and included the “three horizontal and two vertical” corridor axes, which formed a network layout and increased the spatial connectivity among ecological sources. The construction of ecological corridors in the northwest Shanxi should be strengthened to resist sandstorms. Meanwhile, the blocking effect of coal mines on ecological corridors needed to be restored through targeted policies and measures. These results not only provided scientific guidance for the governments to carry out ecological restoration and biodiversity protection, but also gave a classic paradigm for global ecological security maintenance and ecological sustainability.

## Figures and Tables

**Table 1:** Ecological regions and ecological subregions of Shanxi Province.

	Ecological Regions		Ecological Subregions
R-A	Loess Plateau agricultural and grassland ecological region	SR-A1	Northern Shanxi mountains and hills semi-arid grassland ecological subregion
		SR-A2	South central parts of Northern Shaanxi-Western Shanxi loess hills and gullies ecological subregion
		SR-A3	Lvliang Mountain deciduous broad-leaf forest ecological subregion
R-B	Fenwei River Basin agro-ecological region	SR-B1	Fen River Valley agro-ecological subregion
R-C	Yanshan-Taihang Mountains deciduous broad-leaved forest ecological region	SR-C1	Yongding River upper intermountain basin forest, agriculture and grass ecological subregion
		SR-C2	Taihang Mountain deciduous broad-leaved forest ecological subregion
		SR-C3	Taiyue Mountain Hilly deciduous broad-leaf forest ecological subregion
		SR-C4	Taihang Mountain and Taiyue Mountain inter-mountain basin hilly agro-ecological subregion
		SR-C5	Zhongtiao Mountain Hill deciduous broad-leaf forest ecological subregion

**Table 2:** The descriptions of ecosystem service models.

ESs	Model	Model Descriptions	References
WC	InVEST (Water Yield)	Water yield is calculated based on the balance equation of water quantity, and WC can be calculated by combining water yield with runoff coefficient, terrain index, and soil saturated hydraulic conductivity.	[40]
SC	InVEST (Sediment Delivery and Retention)	Including two parts: soil erosion reduction and sediment retention. The former is the difference between potential and actual soil erosion (soil erosions are calculated by the modified soil loss equation), and the latter is the product of sediment and sediment retention rate.	[41,42]
SF	National Wind Erosion Survey Model of China (NWESMC)	SF is the difference between potential and actual soil wind erosion, and the soil wind erosion is estimated by the NWESMC. This model is developed for grassland (forest land), sandy land and farmland, and the parameters are calibrated by the wind tunnel experiments on chestnut-calcium soils and wind-sand soils in a typical semi-arid grassland region of China.	[43]
CS	InVEST (Carbon)	Total CS is the sum of average carbon density of above-ground carbon pool, below-ground carbon pool, and soil carbon pool for different land use types.	[44,45]
NPP	Carnegie Ames Stanford Approach (CASA)	NPP of vegetation is estimated by multiplying absorption photosynthetically active radiation and light energy utilization rate absorbed by vegetation.	[46]
HQ	InVEST (Habitat Quality)	HQ is assessed according to the impact distance and spatial weighting of threat sources, habitat suitability and its sensitivity to threat sources, and access for legal protection.	[47,48]

**Table 3:** Ecosystem services of different land use types.

Land Use Type	WC/mm	SC/t·hm^−2^	SF/t·hm^−2^	CS/t·hm^−2^	NPP/gC·m^−2^	HQ
Farmland	10.10	12.42	1.059	140.3	507.5	0.4940
Forest land	19.35	34.93	4.698	289.3	1029	0.8374
Grassland	14.12	23.11	6.541	153.3	466.5	0.6831
Wet land	7.540	14.45	2.071	0.000	451.5	0.5630
Construction land	9.379	8.135	1.022	66.90	417.6	0.3315
Unused land	9.386	11.68	4.014	27.10	488.5	0.4646

**Table 4:** Ecosystem services in different ecological regions and subregions.

Ecological Regions	Ecological Subregions	WC/mm	SC/t·hm^−2^	SF/t·hm^−2^	CS/t·hm^−2^	NPP/gC·m^−2^	HQ
R-A	SR-A1	13.05	5.018	1.912	165.6	560.3	0.5726
	SR-A2	13.43	33.16	0.5763	161.5	522.2	0.6532
	SR-A3	17.00	31.32	0.4279	210.1	832.5	0.7605
R-B	SR-B1	5.547	4.525	0.1123	130.9	439.0	0.3378
R-C	SR-C1	14.09	16.82	13.97	186.0	615.1	0.6917
	SR-C2	14.79	30.31	11.81	187.5	695.0	0.6941
	SR-C3	15.63	23.63	0.7703	199.7	651.4	0.7145
	SR-C4	10.47	11.35	0.3294	157.7	483.7	0.4313
	SR-C5	17.84	34.41	1.471	210.7	736.2	0.7316

## Data Availability

Data sharing not applicable. No new data were created or analyzed in this study. Data sharing is not applicable to this article.

## Supplementary Materials

The following supporting information can be downloaded at: https://www.mdpi.com/article/10.3390/ijerph20064819/s1. References [76,77,78,79,80,81,82,83,84,85,86,87,88,89,90] are cited in the supplementary materials.

## References

[1] Millennium Ecosystem Assessment (2005). Ecosystems and Human Well-Being: Biodiversity Synthesis.

[2] Zhou S., Peng L. (2022). Integrating a mixed-cell cellular automata model and Bayesian belief network for ecosystem services optimization to guide ecological restoration and conservation. Land Degrad. Dev..

[3] Bai Y., Wong C.P., Jiang B., Hughes A.C., Wang M., Wang Q. (2018). Developing China’s Ecological Redline Policy using ecosystem services assessments for land use planning. Nat. Commun..

[4] Yuan M.H., Lo S.L. (2020). Ecosystem services and sustainable development: Perspectives from the food-energy-water Nexus. Ecosyst. Serv..

[5] Hossain M.S., Dearing J.A., Rahman M.M., Salehin M. (2016). Recent changes in ecosystem services and human well-being in the Bangladesh coastal zone. Reg. Environ. Chang..

[6] Sun X., Yang P., Tao Y., Bian H.Y. (2020). Improving ecosystem services supply provides insights for sustainable landscape planning: A case study in Beijing, China. Sci. Total Environ..

[7] Costanza R., d’Arge R., Groot R.D., Farber S., Grasso M., Hannon B., Limburg K., Naeem S., O’Neill R.V., Paruelo J. (1997). The value of the world’s ecosystem services and natural capital. Nature.

[8] Jiang W.G., Deng Y., Tang Z.H., Lei X., Chen Z. (2017). Modelling the potential impacts of urban ecosystem changes on carbon storage under different scenarios by linking the CLUE-S and the InVEST models. Ecol. Model..

[9] Aneseyee A.B., Elias E., Soromessa T., Feyisa G.L. (2020). Land use/land cover change effect on soil erosion and sediment delivery in the Winike watershed, Omo Gibe Basin, Ethiopia. Sci. Total Environ..

[10] Gong J., Cao E., Xie Y.C., Xu C.X., Li H.Y., Yan L.L. (2020). Integrating ecosystem services and landscape ecological risk into adaptive management: Insights from a western mountain-basin area, China. J. Environ. Manag..

[11] Fang Z., Ding T.H., Chen J.Y., Xue S., Zhou Q., Wang Y.X., Huang Z.D., Yang S.L. (2022). Impacts of land use/land cover changes on ecosystem services in ecologically fragile regions. Sci. Total Environ..

[12] Cong W.C., Sun X.Y., Guo H.W., Shan R.F. (2020). Comparison of the SWAT and InVEST models to determine hydrological ecosystem service spatial patterns, priorities and trade-offs in a complex basin. Ecol. Indic..

[13] Wu J.G. (2014). Urban ecology and sustainability: The state-of-the-science and future directions. Landsc. Urban Plan..

[14] Peng J., Zhao H.J., Liu Y.X., Wu J.S. (2017). Research progress and prospect on regional ecological security pattern construction. Geogr. Res..

[15] Harvey E., Gounand I., Ward C.L., Altermatt F. (2017). Bridging ecology and conservation: From ecological networks to ecosystem function. J. Appl. Ecol..

[16] Chen J., Wang S.S., Zou Y.T. (2022). Construction of an ecological security pattern based on ecosystem sensitivity and the importance of ecological services: A case study of the Guanzhong Plain urban agglomeration, China. Ecol. Indic..

[17] Yu K.J. (1999). Landscape ecological security patterns in biological conservation. Acta Ecol. Sin..

[18] Dong J.Q., Peng J., Xu Z.H., Liu Y.X., Wang X.Y., Li B. (2021). Integrating regional and interregional approaches to identify ecological security patterns. Landsc. Ecol..

[19] Dai L., Liu Y.B., Luo X.Y. (2021). Integrating the MCR and DOI models to construct an ecological security network for the urban agglomeration around Poyang Lake, China. Sci. Total Environ..

[20] Wang S., Li W.J., Li Q., Wang J.F. (2022). Ecological security pattern construction in Beijing-Tianjin-Hebei region based on hotspots of multiple ecosystem services. Sustainability.

[21] Ye X., Zou C.X., Liu G.H., Lin N.F., Xu M.J. (2018). Main research contents and advances in the ecological security pattern. Acta Ecol. Sin..

[22] Gao M.W., Hu Y.C., Bai Y.P. (2022). Construction of ecological security pattern in national land space from the perspective of the community of life in mountain, water, forest, field, lake and grass: A case study in Guangxi Hechi, China. Ecol. Indic..

[23] Yang S.S., Zou C.X., Shen W.S., Shen R.P., Xu D.L. (2016). Construction of ecological security patterns based on ecological red line: A case study of Jiangxi Province. Chin. J. Ecol..

[24] Wang X.R., Wan R.R., Pan P.P. (2022). Construction and adjustment of ecological security pattern based on MSPA-MCR Model in Taihu Lake Basin. Acta Ecol. Sin..

[25] Zhang Q., Wang G., Yuan R.Y., Singh V.P., Wu W.H., Wang D.Z. (2022). Dynamic responses of ecological vulnerability to land cover shifts over the Yellow river Basin, China. Ecol. Indic..

[26] Zhang Y.H., Wang Z., Hu S.G., Song Z.Y., Cui X.G., Afriyie D. (2022). Spatial and Temporal Evolution and Prediction of the Coordination Level of “Production-Living-Ecological” Function Coupling in the Yellow River Basin, China. Int. J. Environ. Res. Public Health.

[27] Omer A., Ma Z.G., Yuan X., Zheng Z.Y., Saleem F. (2021). A hydrological perspective on drought risk-assessment in the Yellow River Basin under future anthropogenic activities. J. Environ. Manag..

[28] Ning T., Guo X.Y., Rong Y.J., Du S.X., Li C. (2019). Evaluation of soil conservation function importance of ecosystems in Shanxi Province based on RUSLE model. Bull. Soil Water Conserv..

[29] Hao X.J., Zhang H., Xu X.M., Wang L., Cui Y. (2020). Evolution and simulation of land use/land cover pattern in northern Shanxi Province. Acta Ecol. Sin..

[30] Zhu C.C., Gong J.R., Yang B., Zhang Z.H., Wang B., Shi J.Y., Yue K.X., Zhang W.Y. (2021). Changes of windbreak and sand fixation services and the driving factors in the desert steppe, Inner Mongolia. Acta Ecol. Sin..

[31] Ma Y.J., Su Z.Z. (2003). Study on sandy desertification of present situation and development trend in Shanxi province. J. Soil Water Conserv..

[32] Yang Y.H., Shi Y., Sun W.J., Chang J.F., Zhu J.X., Chen L.Y., Wang X., Guo Y.P., Zhang H.T., Yu L.F. (2022). Terrestrial carbon sinks in China and around the world and their contribution to carbon neutrality. Sci. China Life Sci..

[33] Zhou P., Hou H.L., Zhang H., Liu X.J., Tan W.B. (2021). The development prospects and implementation suggestions of increasing soil carbon storage in the context of carbon neutrality. Environ. Prot..

[34] Ahmed N., Ahmad M., Ahmed M. (2021). Combined role of industrialization and urbanization in determining carbon neutrality: Empirical story of Pakistan. Environ. Sci. Pollut. Res. Int..

[35] Yang Y.P., Chen J.J., Ren J.H., Feng Z.H., Zhou G.Q., You H.T., Han X.W. (2022). Construction of ecological security pattern based on the importance of ecological protection—A case study of Guangxi, a karst region in China. Int. J. Environ. Res. Public Health.

[36] Kang Q., Guo Q.X., Ding Y., Zhang Y., Hu Y., Chen S.Y. (2021). Temporal and spatial evolution ang driving factors of productional-living-ecological functions of Shanxi Province during 2005–2018. Bull. Soil Water Conserv..

[37] Han R., Zhang J.J., Zhu W.B., Wang L.Y., Zhang L.J., Zhu L.Q. (2018). Impact of land use change on habitat in the Qihe River Basin of Taihang Mountains. Prog. Geogr..

[38] He J., Shi X.Y., Fu Y.J. (2020). Optimization of ecological security pattern in the source area of Fen River Basin based on ecosystem services. J. Nat. Resour..

[39] Zhao Q.K., Wang Z.G., Zhang G.C., Zhang C., Hu X.L., Zhang S.Y. (2014). Assessment of eco-environment vulnerability in functional regions of soil and water conservation in Shanxi Province. Sci. Soil Water Conserv..

[40] Hu W., Li G., Li Z. (2021). Spatial and temporal evolution characteristics of the water conservation function and its driving factors in regional lake wetlands—Two types of homogeneous lakes as examples. Ecol. Indic..

[41] Gu Y.Y., Lin N.F., Ye X., Xu M.J., Qiu J., Zhang K., Zou C.X., Qiao X.N., Xu D.L. (2022). Assessing the impacts of human disturbance on ecosystem services under multiple scenarios in karst areas of China: Insight from ecological conservation red lines effectiveness. Ecol. Indic..

[42] Liu M.Z., Zhang H.J., Ren H.Y., Pei H.W. (2021). Spatiotemporal Variations of the Soil Conservation in the Agro-pastoral Ecotone of Northern China under Grain for Green Program. Res. Soil Water Conserv..

[43] Yu B.L., Wu W.J., Zhao X.J., Wu E.T., Cai L.Y., Yang F.J. (2016). Benefits of soil wind erosion control of the Beijing-Tianjin Sand Source Control Project in Inner Mongolia. Arid Zone Res..

[44] Deng Y.J., Yao S.B., Hou M.Y., Zhang T.Y., Lu Y.N., Gong Z.W., Wang Y.F. (2020). Assessing the effects of the Green for Grain Program on ecosystem carbon storage service by linking the InVEST and FLUS models: A case study of Zichang county in hilly and gully region of Loess Plateau. J. Nat. Resour..

[45] Tang L.P., Ke X.L., Zhou T., Zheng W.W., Wang L.Y. (2020). Impacts of cropland expansion on carbon storage: A case study in Hubei, China. J. Environ. Manag..

[46] Dai L.D., Zhang Y.L., Ding R.J., Yan Y.G. (2022). Spatiotemporal Distribution and Influencing Factors of the Net Primary Productivity in the Datai Mine in Western Beijing. Sustainability.

[47] Gong J., Xie Y., Cao E., Huang Q.Y., Li H.Y. (2019). Integration of InVEST-habitat quality model with landscape pattern indexes to assess mountain plant biodiversity change: A case study of Bailongjiang watershed in Gansu Province. J. Geogr. Sci..

[48] Yang Z.P., Xu J.W., Fen X.H., Guo M., Ji Y.H., Gao X.J. (2018). Effects of land use change on habitat based on InVEST model in Northeast China. Ecol. Sci..

[49] Manning P., Fons V.D.P., Soliveres S., Allan E., Maestre F.T., Georgina M., Whittingham M.J., Markus F. (2018). Publisher Correction: Redefining ecosystem multifunctionality. Nat. Ecol. Evol..

[50] Shen J.S., Li S.C., Liang Z., Liu L.B., Li D.L., Wu S.Y., Shen J.S., Li S.C., Liang Z., Liu L.B. (2020). Exploring the heterogeneity and nonlinearity of trade-offs and synergies among ecosystem services bundles in the Beijing-Tianjin-Hebei urban agglomeration. Ecosyst. Serv..

[51] Li Q., Li W.J., Wang S., Wang J.F. (2022). Assessing heterogeneity of trade-offs/synergies and values among ecosystem services in Beijing-Tianjin-Hebei urban agglomeration. Ecol. Indic..

[52] Wu W.H., Peng J., Liu Y.X., Hu Y.N. (2017). Tradeoffs and synergies between ecosystem services in Ordos City. Prog. Geogr..

[53] Li Q., Zhou Y., Yi S.Q. (2022). An integrated approach to constructing ecological security patterns and identifying ecological restoration and protection areas: A case study of Jingmen, China. Ecol. Indic..

[54] Yu K.J., Wang S.S., Li D.H., Li C.B. (2009). The function of ecological security patterns as an urban growth framework in Beijing. Acta Ecol. Sin..

[55] Wang J., Gao J., Yuan M.X., Song Q.Y., Li S. (2021). Construction of rural landscape ecological security pattern from the perspective of biological protection—A case study of Houjialou Village, Yong’an Town, Fenxi County, Linfen City, Shanxi Province. Ecol. Sci..

[56] Gou M.M., Li L., Ouyang S., Shu C., Xiao W.F., Wang N., Hu J.W., Liu C.F. (2022). Integrating ecosystem service trade-offs and rocky desertification into ecological security pattern construction in the Daning river basin of southwest China. Ecol. Indic..

[57] Zhang J.X., Cao Y.M., Ding F.S., Wu J., Chang I.-S. (2022). Regional Ecological Security Pattern Construction Based on Ecological Barriers: A Case Study of the Bohai Bay Terrestrial Ecosystem. Sustainability.

[58] Wen H., Deng X.P., Li Y., Wang C., Wang L.R., He D.J., Wu L.Y., You W.B. (2023). Identification and construction of county-level ecological security pattern integrating ecological space: A case study of Wuyishan City. Chin. J. Ecol..

[59] Yu C.L., Liu D., Feng R., Tang Q., Guo C.L. (2021). Construction of ecological security pattern in Northeast China based on MCR model. Acta Ecol. Sin..

[60] Albert C.H., Rayfield B., Dumitru M., Gonzalez A. (2017). Applying network theory to prioritize multispecies habitat networks that are robust to climate and land-use change. Conserv. Biol..

[61] Pascual-Hortal L., Saura S. (2007). Impact of spatial scale on the identification of critical habitat patches for the maintenance of landscape connectivity. Landsc. Urban Plan..

[62] Wang Y.J., Qu Z.Y., Zhong Q.C., Zhang Q., Zhang L., Zhang R., Yi Y., Zhang C.L., Li X.C., Liu J. (2022). Delimitation of ecological corridors in a highly urbanizing region based on circuit theory and MSPA. Ecol. Indic..

[63] Gibson L., Lee T.M., Koh L.P., Brook B.W., Gardner T.A., Barlow J., Peres C.A., Bradshaw C.J.A., Laurance W.F., Lovejoy T.E. (2011). Primary forests are irreplaceable for sustaining tropical biodiversity. Nature.

[64] Escobar H. (2020). Deforestation in the Brazilian Amazon is still rising sharply. Science.

[65] Macedo M.N., DeFries R.S., Morton D.C., Stickler C.M., Galford G.L., Shimabukuro Y.E. (2012). Decoupling of deforestation and soy production in the southern Amazon during the late 2000s. Proc. Natl. Acad. Sci. USA.

[66] López-Carr D., Ryan S.J., Clark M.L. (2022). Global economic and diet transitions drive Latin American and Caribbean Forest change during the first decade of the century: A multi-scale analysis of socioeconomic, demographic, and environmental drivers of local forest cover change. Land.

[67] Samson F., Knopf F. (1994). Prairie conservation in North America. Bioscience.

[68] Valkó O., Rádai Z., Deák B. (2022). Hay transfer is a nature-based and sustainable solution for restoring grassland biodiversity. J. Environ. Manag..

[69] Osland M.J., Hughes A.R., Armitage A.R., Scyphers S.B., Cebrian J., Swinea S.H., Shepard C.C., Allen M.S., Feher L.C., Nelson J.A. (2022). The impacts of mangrove range expansion on wetland ecosystem services in the southeastern United States: Current understanding, knowledge gaps, and emerging research needs. Glob. Chang. Biol..

[70] Mengist W., Soromessa T., Feyisa G.L. (2023). Responses of carbon sequestration service for landscape dynamics in the Kaffa biosphere reserve, southwest Ethiopia. Environ. Impact Assess. Rev..

[71] Anderson R., Bayer P.E., Edwards D. (2020). Climate change and the need for agricultural adaptation. Curr. Opin. Plant Biol..

[72] Valeriy R., Tatyana K., Alexander B., Irina G., Denis D., Tatyana R., Galina S., Ruslan L. (2022). A drastic change in glacial dynamics at the beginning of the seventeenth century on Novaya Zemlya coincides in time with the strongest volcanic eruption in Peru and the Great Famine in Russia. Quat. Res..

[73] Pereira P., Bašić F., Bogunovic I., Barcelo D. (2022). Russian-Ukrainian war impacts the total environment. Sci. Total Environ..

[74] Li Y., Wen T. (2022). Impact of cognition and social trust on forest-based health tourism intention during COVID-19. Sustainability.

[75] Berkenbosch K., Groote P., Stoffelen A. (2022). Industrial heritage in tourism marketing: Legitimizing post-industrial development strategies of the Ruhr Region, Germany. J. Herit. Tour..

[76] Budyko M.I. (1974). Climate and Life.

[77] Liu X.N., Pei X., Chen L., Liu C.L. (2018). Study on soil conservation service of ecosystem based on InVEST model in Mentougou District of Beijing. Res. Soil Water Conserv..

[78] Wang S., Li Y.W., Li Q., Hu S.X., Wang J.F., Li W.J. (2022). Water and soil conservation and their trade-off and synergistic relationship under changing environment in Zhangjiakou-Chengde area. Acta Ecol. Sin..

[79] Wischmeier W.H. (1971). A soil erodibility nomograph for farmland and construction sites. J. Soil Water Conserv..

[80] Williams J.R., Jones C.A., Dyke P.T. (1984). A modeling approach to determining the relationship between erosion and soil productivity. Trans. ASAE.

[81] Dong M. (2020). Evolution of Ecosystem Services and Its Influencing Factors in the Upper Reach of the Fenhe River Basin. M.D. Thesis.

[82] Wang X.Y. (2020). Potential Wind Erosion Simulation Using Different Models in the Agro-Pastoral Ecotone of Northern China. M.D. Thesis.

[83] Liu Y., Zhang J., Zhou D.M., Ma J., Dang R., Ma J.J., Zhu X.Y. (2021). Temporal and spatial variation of carbon storage in the Shule River Basin based on InVEST model. Acta Ecol. Sin..

[84] Tang X.L., Zhao X., Bai Y.F., Tang Z.Y., Wang W.T., Zhao Y.C., Wan H.W., Xie Z.Q., Shi X.Z., Wu B.F. (2018). Carbon pools in China’s terrestrial ecosystems: New estimates based on an intensive field survey. Proc. Natl. Acad. Sci. USA.

[85] Zhou J.J., Zhao Y.R., Huang P., Zhao X., Feng W., Li Q.Q., Xue D.X., Dou J., Shi W., Wei W. (2020). Impacts of ecological restoration projects on the ecosystem carbon storage of inland river basin in arid area, China. Ecol. Indic..

[86] Li K.R., Wang S.Q., Cao M.K. (2003). Carbon storage of vegetation and soil in China. Sci. Chin..

[87] Xue X.Y., Wang X.Y., Duan H.M., Xie Y.W. (2021). Temporal and spatial changes of NPP and its causes in the agricultural pastoral ecotone of Northern China. Res. Soil Water Conserv..

[88] Zhu W.Q., Pan Y.Z., He H., Yu D.Y., Hu H.B. (2006). Simulation of maximum light use efficiency for some typical vegetation types in China. Chin. Sci. Bull..

[89] Chen Y.Q., Zhao L., Tao J.Y., Zhang P.T. (2020). Habitat quality evaluation before and after unused land development based on the InVEST model: A case study of Tang Country. Chin. J. Eco-Agric..

[90] He J., Shi X.Y., Fu Y.J., Zhang Y. (2020). Multi-scenario simulation of spatiotemporal evolution of land use and habitat quality in the source area of Fenhe River Basin. Res. Soil Water Conserv..

