# Supplementary material for: Assessment of Multiple Ecosystem Services and Ecological Security Pattern in Shanxi Province, China
Source: Int J Environ Res Public Health. 2023 Mar 9;20(6):4819. doi: 10.3390/ijerph20064819 (PMC10049408; doi:10.3390/ijerph20064819)
Supplement: Supplementary file 1 [file ijerph-20-04819-s001.zip › ijerph-2231117-supplementary.pdf]

Supplementary Materials

File S1

Table S1. The terms used in this study and their abbreviations

Terms	Abbreviations	Terms	Abbreviations
Advanced Spaceborne Thermal Emission and Reflection Radiometer Global Digital Elevation Model	ASTER GDEM	Land Use/Land Cover	LULC
Analytic Hierarchy Process	AHP	Minimum Cumulative Resistance	MCR
Carbon Storage	CS	Multiple Ecosystem Services Landscape Index	MESLI
Carnegie Ames Stanford Approach	CASA	National Aeronautics and Space Administration	NASA
Digital Elevation Model	DEM	National Climatic Data Center	NCDC
Duranton-Overman Index	DOI	National Wind Erosion Survey Model of China	NWESMC
Ecosystem Services	ESs	Net Primary Productivity	NPP
Geographic Information System	GIS	Normalized Difference Vegetation Index	NDVI
Habitat Quality	HQ	Sand Fixation	SF
Integrated Valuation of Ecosystem Services and Tradeoffs	InVEST	Soil Conservation	SC
Kaiser-Meyer-Olkin	KMO	Water Conservation	WC

File S2: Methods

(1) Water Conservation (WC)

Water conservation (WC) is calculated by combining water yield with runoff coefficient, terrain index, and soil saturated hydraulic conductivity [1–3]. The formula is as follows:

$$WC = \min\left(1, \frac{249}{V}\right) \times \min\left(1, 0.9 \times \frac{TI}{3}\right) \times \min\left(1, \frac{K}{300}\right) \times Y$$

Where WC is annual water conservation (mm), V is runoff coefficient, TI is terrain index, K is soil saturated hydraulic conductivity (cm/d), and Y is annual water yield (mm).

Based on the balance equation of water quantity, water yield is estimated by the water yield module in the InVEST model. The formulas are as follows:

$$Y_{xj} = \left(1 - \frac{AET_{xj}}{P_x}\right) \times P_x$$

$$\frac{AET_{xj}}{P_x} = \frac{1 + \omega_x R_{xj}}{1 + \omega_x R_{xj} + \frac{1}{R_{xj}}}$$

$$\omega_x = \frac{AWC_x}{P_x} \times Z$$

$$R_{\mathrm{xj}} = \frac{K_{\mathrm{xj}} \times ET_{ox}}{P_x}$$

Where Y_{xj} (mm) is the annual water yield of land use type j in grid x; AET_{xj} (mm) is the annual actual evapotranspiration of land use type j in grid x; P_x (mm) is the annual precipitation in grid x; R_{xj} is Budyko dryness index [1], which is the ratio of annual potential evapotranspiration (ET_{ox}) to annual precipitation (P_x); ω_x is a dimensionless parameter, representing the ratio of annual vegetation water requirement to annual precipitation. AWC_x (mm) is the effective water content of plants, which is obtained by a nonlinear model [3]. Z ($0 \le Z \le 30$) is the seasonal constant, which is obtained by the total amount of surface water resources from the Water Resources Bulletin of Shanxi Province (Table S2).

Table S2. Water resources of Shanxi Province in 2020.

**Year**	**Total area ($\times 10^4 km^2$)**	**Total water resources ($\times 10^8 m^3$)**	**Surface water volume ($\times 10^8 m^3$)**	**Groundwater volume ($\times 10^8 m^3$)**	**Duplicated volume ($\times 10^8 m^3$)**	**Water yield (mm)**	**Z**
2020	15.67	115.15	72.21	85.92	42.98	73.69	4.65

(2) Soil Conservation (SC)

Soil Conservation (SC) is estimated by the Sediment delivery and retention (SDR) module in the InVEST model. The calculation can be divided into two parts: soil erosion reduction and sediment retention. The soil erosion reduction is the difference between potential soil erosion and actual soil erosion, and the sediment retention is the product of sediment and sediment retention rate [4]. The formulas are as follows:

$$SEDRET_x = R_x \times K_x \times LS_x \times (1 - C_x \times P_x) + SEDR_x$$

$$SEDR_x = SE_x \sum_{y=1}^{x-1} USLE_y \prod_{z=y+1}^{x-1} (1 - SE_z)$$

$$USLE_x = R_x \times K_x \times LS_x \times C_x \times P_x$$

Where $SEDRET_x$ and $SEDR_x$ are the soil conservation and sediment retention of grid x, respectively; $USLE_x$ and $USLE_y$ are the actual soil erosion of grid x and its uphill grid y, respectively; SE_x stands for the sediment retention rate of grid x; R_x is rainfall erosivity factor (MJ mm hm^{-2} h^{-1} a^{-1}), is calculated by using the Wischmeier formula based on monthly and

annual precipitation [5]; K_x is the soil erodibility factor (t h MJ^{-1} mm^{-1}), is calculated by the formula established by Williams et al [6]; LS_x is the slope length factor; C_x and P_x are vegetation management factor, and soil and water conservation measures factor, respectively, which are obtained by the relevant studies (Table S3) in Shanxi Province [7].

Table S3. Vegetation management factor (C) and soil and water conservation factor (P) for different land use types.

LULC / Factors	Farmland	Forest land	Grassland	Wet land	Construction land	Unused land
C	0.35	0.09	0.3	0	0	0.7
P	0.15	0.9	1	0	1	1

(3) Sand Fixation (SF)

Sand fixation (SF) is equal to the potential soil wind erosion (soil wind erosion without vegetation cover) minus the actual soil wind erosion (soil wind erosion with actual vegetation cover), and the formula is:

$$G = Q_{pot} - Q_{act}$$

Where *G* (t) is the annual sand fixation; Q_{pot} (t) is the annual potential soil wind erosion; Q_{act} (t) is the annual actual soil wind erosion.

Considering climate, land use, vegetation cover and surface roughness, the soil wind erosion is estimated by the National Wind Erosion Survey Model of China (NWESMC) [8]. This model is developed for different land use types, and the parameters are calibrated by the wind tunnel experiments on chestnut-calcium soils and wind-sand soils in a typical semi-arid grassland region of China [9]. The formulas of soil wind erosion for grassland (forest land), sandy land and farmland are as follows:

$$Q_{fa} = 10 \cdot \hat{C} \cdot \sum_{j=1} \left\langle T_j \cdot \exp\left\{ a_1 + \frac{b_1}{z_0} + c_1 \cdot \left[\left(A \cdot U_j \right)^{0.5} \right] \right\} \right\rangle$$

$$Q_{fgf} = 10 \cdot \hat{C} \cdot \sum_{j=1} \left\{ T_j \cdot \exp\left[a_2 + b_2 \cdot VC^2 + c_2 / \left(A \cdot U_j \right) \right] \right\}$$

$$Q_{fs} = 10 \cdot \hat{C} \cdot \sum_{j=1} \left\{ T_j \cdot \exp\left[a_3 + b_3 \cdot VC + c_3 \cdot \ln\left(A \cdot U_j \right) / \left(A \cdot U_j \right) \right] \right\}$$

Where Q_{fa}, Q_{fgf} and Q_{fs} (t hm^{-2}) are soil wind erosion modulus for the farmland, grassland (forest land) and sandy land, respectively; *VC* (%) is the vegetation coverage, if *VC*=0, the Q_{fgf} and Q_{fs} are potential soil wind erosion modulus, if VC is the actual vegetation coverage, Q_{fgf} and Q_{fs} are actual soil wind erosion modulus; U_j is the j-level wind speed (m/s) higher than the critical erosion wind speed (5m s^{-1}); T_j is the cumulative time (min) of the j-level wind speed; *C* and *A* are two correction coefficients, *C*=0.0018, and the value of *A* is related to the underlying surface; a_1, b_1, c_1, a_2, b_2, c_2, a_3, b_3 and c_3 are constants, whose values are -9.208, 0.018, 1.955, 2.4869, -0.0014, -54.9472, 6.1689,-0.0743 and -27.9613, respectively [8-9].

(4) Carbon Storage (CS)

Carbon storage (CS) of terrestrial ecosystem includes four carbon pools: aboveground biomass, belowground biomass, soil and dead organic matter [10]. In this study, the carbon density of dead organic matter is ignored due to its small value. The CS is the sum of rest three carbon pools, and the formula is as follows:

$$C_i = C_{i-above} + C_{i-below} + C_{i-soil}$$

$$C_{tot} = \sum_{i=1}^{n} C_i \times S_i$$

Where C_i (t hm^{-2}) is the carbon density for a certain land use type i; $C_{i\text{-}above}$ (t hm^{-2}) is the aboveground carbon density, refers to the biomass of all living vegetation above the soil layer expressed by dry weight, including stems, piles, branches, etc; $C_{i\text{-}below}$ (t hm^{-2}) is the belowground carbon density, encompasses the living root systems of aboveground biomass. $C_{i\text{-}soil}$ (t hm^{-2}) is the soil carbon density, which is the organic component of soil (including fine roots). C_{tot} (t) is the total carbon storage of terrestrial ecosystem, S_i (hm^2) is the area of a certain land use type (i), n (n=6) is the number of land use types.

Biomass and soil carbon density are negatively correlated with precipitation, and positively correlated with air temperature [11]. Therefore, air temperature and precipitation data can be used to correct the initial carbon density data (such as the carbon density of China) to obtain the local data (such as data of Shanxi province) [12]. The multi-year average temperature and precipitation are 7.56°C and 673.9mm in China, and are 10.5°C and 547.1mm in Shanxi province. According to the method proposed by Zhou et al. [12], the correction coefficient of biomass or soil carbon density between Shanxi province and China could be calculated. The correction coefficient of biomass carbon density was 0.57, and the correction coefficient of soil carbon density was 0.93. Finally, the carbon density of Shanxi province was estimated by the product of carbon density in the whole China [11,13] and above two corrected coefficients (Table S4).

Table S4. Carbon density in Shanxi province (t hm^{-2}).

Land use	Aboveground biomass	Belowground biomass	Soil
Farmland	3.25	45.99	91.06
Forest land	24.16	66.06	198.99
Grassland	20.12	49.31	83.92
Wet land	0	0	0
Construction land	1.43	0	65.52
Unused land	0.74	0	26.38

(5) Net Primary Productivity (NPP)

According to the principle of Carnegie-Ames-Stanford Approach (CASA) model [14], Net primary productivity (NPP) of vegetation is estimated by simulating light and effective radiation of vegetation and actual light energy utilization rate [15]. The formulas are as follows:

$$NPP_{(x,t)} = APAR_{(x,t)} \times \varepsilon_{(x,t)}$$

$$APAR_{(x,t)} = 0.5 \times SOL_{(x,t)} \times FPAR_{(x,t)}$$

Where $APAR_{(x,\ t)}$ is the photosynthetically active radiation absorbed of grid x in month t; $\varepsilon_{(x,\ t)}$ is the light energy utilization rate of grid x in month t; $SOL_{(x,\ t)}$ is the total solar radiation of grid x in month t, $FPAR(x,\ t)$ is the absorption ratio of incident photosynthetically active radiation by the vegetation layer of grid x in month t, and constant 0.5 is the proportion of

solar effective radiation available for vegetation to the total solar radiation.

(6) Habitat Quality (HQ)

Habitat quality (HQ) refers to the suitability assessments by combining the habitat factors in the ecosystem, human survival with social-economic sustainable development in a specific space-time range. In the habitat quality module of InVEST model, the habitat quality index is calculated to comprehensively assess ecosystem services through considering the influence distance, weight and sensitivity of threat factors [16]. The formula is as follows:

$$Q_{xj} = H_j \left[1 - \left(\frac{D_{xj}^Z}{D_{xj}^Z + k^Z} \right) \right]$$

Where Q_{xj} is the habitat quality index of grid x in land use type j; D_{xj} is the threat level of grid x in land use type j; K is the semi saturation constant; H_j is the habitat suitability of land use type j; Z is a normalized constant, usually taking the default parameter value of 2.5.

According to the land use status of Shanxi Province, construction land, farmland and unused land related to human activities are selected as threat factors. Referring to the existing studies [17], the values of threat factor, sensitivity, influence distance and weight are assigned (Table S5 and Table S6).

Table S5. Threat factors and weight in Shanxi Province.

Threat type	Max distance	Weight	Decay
Construction land	4	0.6	Linear
Farmland	8	0.9	Exponential
Unused land	2	0.2	Linear

Table S6. The habitat suitability and sensitivity of land use type to each threat factor.

Threat type	Habitat suitability	Construction land	Farmland	Unused land
Farmland	0.3	0.4	0.4	0
Forest land	1	0.8	0.8	0.2
Grassland	0.8	0.7	0.7	0.5
wetland	0.9	0.5	0.9	0.2
Construction land	0	0	0	0
Unused land	0.2	0.2	0.3	0

File S3

Table S7. Resistance factors class and the weight for ecological source.

Resistance Factor	Weight	Resistance Class	value	Resistance Class	value	Resistance Class	value
River	0.1024	<0.5km	1	0.5-1km	3	1-2km	5
		2-5km	7	>5km	9		
National Highway	0.0492	<0.5km	9	0.5-1km	7	1-2km	5
		2-5km	3	>5km	1	>10	0
Provincial Highway	0.0394	<0.25km	9	0.25-0.5km	7	0.5-1km	5
		1-2km	3	2-5km	1	>5km	0
Highway	0.0302	<1km	9	1-2km	7	2-5km	5
		5-10km	3	10-15km	1	>15km	0
Railway	0.0192	<1km	9	1-2km	7	2-5km	5
		5-10km	3	10-15km	1	>15km	0

DEM	0.0341	<0.8km	9	0.8-1km	7	1-1.5km	5
		1.5-2km	3	>2km	1		
Slope	0.1005	<5°	9	5-8°	7	8-15°	5
		15-20°	3	>20°	1		
NDVI	0.0930	0-0.2	9	0.2-0.4	7	0.4-0.6	5
		0.6-0.7	3	>0.7	1		
ESs	0.4318	Extremely	1	Highly	3	Moderately	5
		Relatively	7	Generally	9		
LULC	0.1002	Farmland	5	Forest land	0	Grassland	1
		Wetland	3	Construction land	9	Unused land	9

Table S8. Resistance factors class and the weight for urban land.

Resistance Factor	Weight	Resistance Class	value	Resistance Class	value	Resistance Class	value
River	0.0340	<0.5km	9	0.5-1km	7	1-2km	5
		2-5km	3	>5km	1		
National Highway	0.2710	<0.5km	0	0.5-1km	1	1-2km	3
		2-5km	5	>5km	7	>10	9
Provincial Highway	0.2209	<0.25km	0	0.25-0.5km	1	0.5-1km	3
		1-2km	5	2-5km	7	>5km	9
Highway	0.0904	<1km	0	1-2km	1	2-5km	3
		5-10km	5	10-15km	7	>15km	9
Railway	0.0707	<1km	0	1-2km	1	2-5km	3
		5-10km	5	10-15km	7	>15km	9
DEM	0.0316	<0.8km	1	0.8-1km	3	1-1.5km	5
		1.5-2km	7	>2km	9		
Slope	0.0949	<5°	1	5-8°	3	8-15°	5
		15-20°	7	>20°	9		
NDVI	0.0305	0-0.2	1	0.2-0.4	3	0.4-0.6	5
		0.6-0.7	7	>0.7	9		
ESs	0.1006	Extremely	9	Highly	7	Moderately	5
		Relatively	3	Generally	1		
LULC	0.0554	Farmland	3	Forest land	9	Grassland	7
		Wetland	5	Construction land	0	Unused land	1

Table S9. The area percentage (%) of ecosystem service hotspots in different regions of Shanxi Province.

Ecological regions	Ecological subregions	0	1	2	3	4	5	6
	SR-A1	29.20	29.26	21.32	11.10	7.39	1.63	0.10
R-A	SR-A2	9.62	28.44	33.88	19.11	5.99	2.92	0.03
	SR-A3	6.12	15.03	19.86	19.43	20.62	18.89	0.04
R-B	SR-B1	78.17	14.67	4.65	1.70	0.67	0.15	0
	SR-C1	17.96	14.85	16.24	16.72	15.89	12.09	6.23
	SR-C2	13.90	13.97	16.06	18.99	18.98	13.90	4.20
R-C	SR-C3	15.16	20.79	19.90	16.89	16.44	10.82	0
	SR-C4	52.29	20.20	11.68	7.75	5.78	2.22	0.09
	SR-C5	18.30	16.10	11.03	10.20	19.77	22.63	1.98

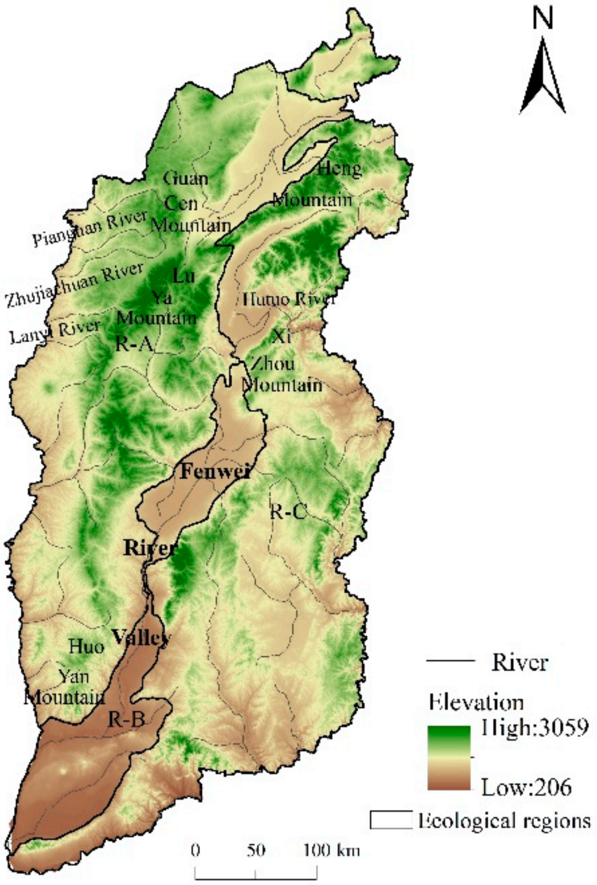

Figure S1. The main mountains and rivers in Shanxi Province.

References

1. Budyko, M.I. Climate and life. Academic: San Diego, California, 1974.
2. Liu, X.N.; Pei, X.; Chen, L.; Liu, C.L. Study on soil conservation service of ecosystem based on InVEST model in Mentougou District of Beijing. *Res. Soil Water Conserv.* **2018,** *25*, 168-176. http://doi.org/10.13869/j.cnki.rswc.2018.06.025.
3. Wang, S.; Li, Y.W.; Li, Q.; Hu, S.X.; Wang, J.F.; Li, W.J. Water and soil conservation and their trade-off and synergistic relationship under changing environment in Zhangjiakou-Chengde area. *Acta Ecol. Sin.* **2022,** *42,* 5391-5403. http://doi.org/10.5846 /stxb202105291417.
4. Liu, M.Z.; Zhang, H.J.; Ren, H.Y.; Pei, H.W. Spatiotemporal variations of the soil conservation in the Agro-pastoral ecotone of northern China under grain for green program. *Res. Soil Water Conserv.* **2021**, *28,* 172-178. http://doi.org/10.13869/j.cnki.rswc.2021.05.020.
5. Wischmeier, W.H. A soil erodibility nomograph for farmland and construction sites. *J. Soil Water Conserv.* **1971**, *26*, 189-193. http://doi.org/10.2307/3896643.
6. Williams, J.R.; Jones, C.A.; Dyke, P.T. A modeling approach to determining the relationship between erosion and soil productivity. *Trans. ASAE,* **1984**, *27*, 129-144. http://doi.org/10.13031/2013.32748.
7. Dong, M. Evolution of ecosystem services and its influencing factors in the upper reach of the Fenhe River Basin. M.D. Thesis, The Shanxi University, Taiyuan, China, 2020.
8. Wang, X.Y. Potential wind erosion simulation using different models in the agro-pastoral ecotone of northern China. M.D. Thesis, Hebei Normal University, Shijiazhuang, China, 2020.
9. Yu, B.L.; Wu, W.J.; Zhao, X.J.; Wu, E.T.; Cai, L.Y.; Yang, F.J. Benefits of soil wind erosion control of

the Beijing-Tianjin Sand Source Control Project in Inner Mongolia. *Arid Zone Res.* **2016**, *33*, 1278-1286. http://doi.org/10.13866/j.azr.2016.06.19.

10. Liu, Y.; Zhang, J.; Zhou, D.M.; Ma, J.; Dang, R.; Ma, J.J.; Zhu, X.Y. Temporal and spatial variation of carbon storage in the Shule River Basin based on InVEST model. *Acta Ecol. Sin*. **2021**, *41*, 4052-4065. https://doi.org/10.5846/stxb201911152452.
11. Tang, X.L.; Zhao, X.; Bai, Y.F.; Tang, Z.Y.; Wang, W.T.; Zhao, Y.C.; Wan, H.W.; Xie, Z.Q.; Shi, X.Z.; Wu, B.F.; et al. Carbon pools in China's terrestrial ecosystems: new estimates based on an intensive field survey. *Proc. Natl. Acad. Sci. USA* **2018**, 115, 4021-4026. https://doi.org/10.1073/pnas.1700291115.
12. Zhou, J.J.; Zhao, Y.R.; Huang, P.; Zhao, X.; Feng, W.; Li, Q.Q.; Xue, D.X.; Dou, J.; Shi, W.; Wei, W.; et al. Impacts of ecological restoration projects on the ecosystem carbon storage of inland river basin in arid area, China. *Ecol. Indic.* **2020**, 118, 106803. https://doi.org/10.1016/j.ecolind.2020.106803.
13. Li, K.R.; Wang, S.Q.; Cao, M.K. Carbon storage of vegetation and soil in China. *Sci. Chin.* **2003**, *33*, 72-80. https://doi.org/10.3321/j.issn:1006-9267.2003.01.008.
14. Xue, X.Y.; Wang, X.Y.; Duan, H.M.; Xie, Y.W. Temporal and spatial changes of NPP and its causes in the agricultural pastoral ecotone of Northern China. *Res. Soil Water Conserv.* **2021**, *28*, 190-197. http://doi.org/10.13869/j.cnki.rswc.2021.02.027.
15. Zhu, W.Q.; Pan, Y.Z.; He, H.; Yu, D.Y.; Hu, H.B. Simulation of maximum light use efficiency for some typical vegetation types in China. *Chin. Sci. Bull.* **2006**, *51*, 457–463. https://doi.org/10.1007/s11434-006-0457-1.
16. Chen, Y.Q; Zhao, L.; Tao, J.Y.; Zhang, P.T. Habitat quality evaluation before and after unused land development based on the InVEST model: a case study of Tang Country. *Chin. J. Eco-Agric.* **2020**, *28*, 1093-1102. https://doi.org/10.13930/j.cnki.cjea.190862.
17. He, J.; Shi, X.Y.; Fu, Y.J.; Zhang, Y. Multi-scenario simulation of spatiotemporal evolution of land use and habitat quality in the source area of Fenhe River Basin. *Res. Soil Water Conserv.* **2020**, *27*, 250-258. https://doi.org/10.13869/j.cnki.rswc.2020.05.033.
